# Supplementary material for: A Proteomic Examination of Plasma Extracellular Vesicles Across Colorectal Cancer Stages Uncovers Biological Insights That Potentially Improve Prognosis
Source: Cancers (Basel). 2024 Dec 21;16(24):4259. doi: 10.3390/cancers16244259 (PMC11674649; doi:10.3390/cancers16244259)
Supplement: Supplementary file 1 [file cancers-16-04259-s001.zip › Supplementary Figure S1.pdf]

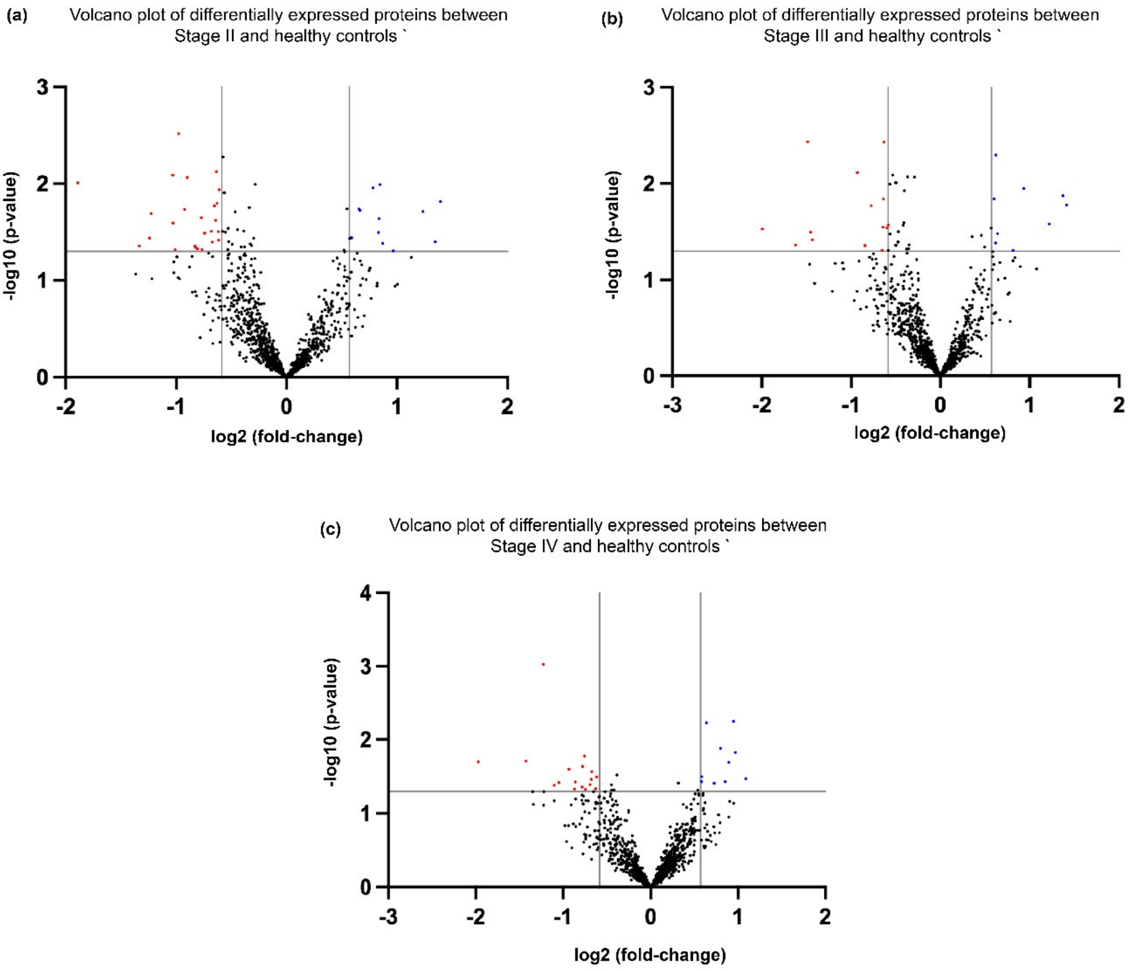

Supplementary Figure S1: Plasma EV protein quantification on CRC stage II, stage III, stage IV vs. healthy controls (a - c). Volcano plot representations on differentially expressed proteins (FC > 1.5, p-value < 0.05) between stages II, III, IV and healthy controls. Blue dots indicate up-regulated proteins and red dots indicate down-regulated proteins.

**CRC stage II vs Healthy control**	
**Up-regulated**	**Down-regulated**
Q13200	P52565
P30498	Q9NTJ5
P49721	P23229
P02786	Q9H939
P01009	P30086
Q96BY6	P40926
P02750	Q4KMP7
P00738	Q13884

**CRC stage III vs Healthy control**	
**Up-regulated**	**Down-regulated**
P04439	P40926
P33151	Q7LDG7
P30498	P11234
P00740	Q08431
P36959	Q01813
P16452	Q9Y696
P54709	P07947
O94804	P00739

**CRC stage IV vs Healthy control**	
**Up-regulated**	**Down-regulated**
P49721	P09382
P05387	P04156
Q9H444	P07947
Q96DZ9	P10909
P05164	Q9H0E2
Q9ULH1	P02652
Q15185	O75131
P54709	P17858

Q9H444	P54920
Q9ULH1	P07951
P05387	P01903
Q06033	P35998
	Q08431
	Q9UHB6
	Q14554
	P14406
	Q99798
	P26927
	P35813
	P83593
	P01033
	Q9ULL4
	P35609
	P02792
	Q9BXJ4
	Q15848

B2RUZ4	Q9UDY2
	Q99798
	Q86WR7
	P01701
	P01701
	P35609
	Q15485
	Q14012

P51809	P41226
	P06727
	P32119
	Q15762
	P02655
	P02747
	Q16543
	P28482
	P02745
